# Fatty acid addition strategy redirected the metabolic flux towards an ultra-high monensin productivity of *Streptomyces cinnamonensis*

**DOI:** 10.1016/j.synbio.2025.02.009

**Published:** 2025-02-18

**Authors:** Shanfei Zhang, Qingming Hou, Zhenhua Wang, Dandan Tian, Xianyuan Zhang, Yan Zhang, Qun Wu, Fubao Sun

**Affiliations:** a Key Laboratory of Industrial Biotechnology, Ministry of Education, School of Biotechnology, Jiangnan University, Wuxi, 214122, China; b Shandong Shengli Bioengineering Co., Ltd., Jining, 272000, China

**Keywords:** Carbon flux redirection, Fatty acid, Secondary metabolites, Polyketide, *Streptomyces cinnamonensis*

## Abstract

Monensin, a polyether ionophore antibiotic produced by *Streptomyces cinnamonensis*, exhibits notable anticoccidial and antitumor properties. In this study, a fatty acid addition (FAA) strategy significantly enhanced the monensin production capability of *S*. *cinnamonensis*, resulting in an unprecedented monensin titer of 17.72 g/L at 192 h, 7.36 times that of the control. Physiological assay showed the FAA markedly altered the cellular morphology, cell membrane fluidity, enzymatic activity and intracellular cofactors, thus indicating of an increased carbon flux. With transcriptional analysis at the product biosynthesis phase, 4 genes in the monensin biosynthesis cluster and 11 genes related to the oxidative stress response were observed to be upregulated. Meanwhile, genes consisting of two sugar transport systems were downregulated. For the precursors supply, genes associated with triacylglycerols (TAG) degradation (*lps*) and fatty acid degradation genes (*fadE*, *fadB*, *fadA*) were upregulated, while genes to TAG synthesis were downregulated. For the monensin synthetic pathway, 8 polyketide synthase genes, 9 modifier genes and 3 pathway-specific regulatory genes within the monensin biosynthetic gene cluster (*mon*) were upregulated. Consequently, the physiological and transcriptional response of *S. cinnamonensis* to the FAA strategy was correlated well with the monensin biosynthesis. The findings not only elucidated the *de novo* biosynthesis of monensin via FAA, but also offered a strategic framework for efficient production of polyketide natural products.

## Graphical abstract

**Figure:**
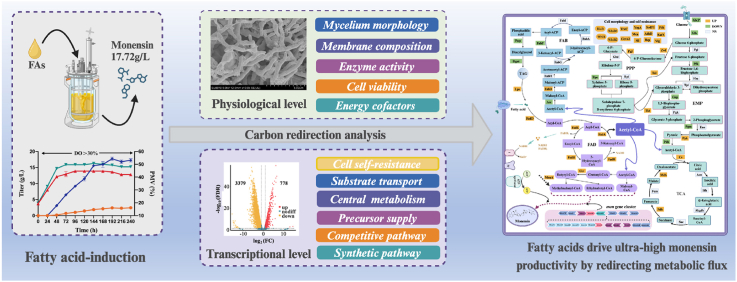

## 1 Introduction

Monensin, a pentacyclic monocarboxylic acid polyether antibiotic that is synthesized by *Streptomyces cinnamonensis,* is generally accepted for its high efficacy, low toxicity, and minimal resistance [1,2]. Accordingly, the monensin has emerged as a preferential anticoccidial agent in the feed industry, i.e., livestock and poultry as it can effectively combat the coccidiosis [3,4]. Recent studies have unveiled a broad spectrum of antitumor for the monensin, thus rendering it to be a potential novel therapeutic in oncology [5,6]. Therefore, the diverse applications of monensin in Veterinary medicine and human health have driven significant research attention to microbial fermentation for monensin production.

Primary challenge in the monensin production lies in the low titer, together with the high cost associated with the process. In response, dozens of researchers have explored diverse strategies to enhance the monensin titer. Tang et al. [7] and Zhang et al. [8] increased monensin titers to 1.13 g/L and 1.33 g/L, respectively, through the tandem overexpression of genes within the monensin synthesis cluster, including *monRI*, *monRII* and *dasR*. Lin et al. achieved a monensin titer of 0.84 g/L by overexpressing the global transcription factor cyclic AMP receptor protein (Crp) that regulates the monensin biosynthesis [9]. Additionally, Liu et al. enhanced the monensin titer by 124.6 %, reaching 1.29 g/L, through the heterologous expression of a polyether-specific transporter SLNHY_0929 [10]. Despite these advancements, the achieved titers have not yet met the requirements for industrial-scale production. Besides metabolic engineering of microbial strains, there have been a lot of efforts in process optimization and control of the microbial monensin fermentation. Considering the common use of vegetable oil as the main source of fatty acids in antibiotic fermentation, we previously investigated the effect of vegetable oil on monensin biosynthesis by *S. cinnamonensis*, finding that various vegetable oil, with varying compositional ratio of oleic, linoleic, stearic, and palmitic acids, exhibited a positive role on monensin production to some different degree. With a further optimization of these four major fatty acids, the addition of formulated fatty acids boosted the monensin production by above 20 %, resulting in a high titer of 15 g/L at a shake-flask level (To be reported elsewhere). However, it is still unclear about the underlying mechanism behind the boosted monensin biosynthesis contributed by fatty acids addition (FAA) strategy.

Actually, considerable efforts have been exerted in investigating the biosynthetic mechanism of monensin. Initially, Day et al. revealed that monensin A is derived from five acetate, seven propionate, and one butyrate precursors, through carbon isotope labeling [11]. In a pivotal study, a gene cluster essential for the monensin biosynthesis was identified in *S. cinnamonensis* [12], and it encodes tailoring enzymes MonBI, MonBII, and MonCI implicated in the oxidative cyclization of linear polyketide intermediates [13,14]. With partial elucidation of the biosynthetic monensin mechanism, it is widely recognized to date that the monensin biosynthesis and fatty acid biosynthesis shared the common precursors, chemical compositions, structural similarities, and an analogous architectural framework [15]. Yet, the precise interaction between these two biosynthetic routes remains obscure. This complexity is often intensified by significant morphological and physiological transformations that are not fully understood, reflecting a metabolic shift from primary central carbon metabolism to specialized secondary metabolism [16,17]. In summary, there still remains a big gap in understanding it's *de novo* synthesis. The biosynthetic pathway of monensin and the reason why fatty acids promote the ultra-high production of monensin need to be further studied.

This study delved into the mechanism that the FAA strategy expedited metabolic transformation and enhanced the monensin production in *S. cinnamonensis*. Initially, the physiological response of *S. cinnamonensis* to fatty acids was assessed, focusing on mycelium morphology, membrane composition, enzymatic activity, cellular viability, and energy cofactor levels. Subsequently, a comparative transcriptomic analysis was performed to elucidate the molecular mechanisms associated with increased monensin production, encompassing aspects such as cell morphology, self-resistance, substrate transport, central carbon metabolism, precursor supply, competitive pathways and synthetic pathways. Ultimately, the metabolic processes through which fatty acids stimulate monensin biosynthesis in *S. cinnamonensis* were elucidated. This research not only provided novel insights into the *de novo* synthesis mechanism of monensin but also charted a new course for the high-yield production of polyketide natural products.

## 2 Materials and methods

### 2.1 Strains and the culture with FAA

Strain *S. cinnamonensis* M6002 was deposited in the China General Microbiological Culture Collection Center (CGMCC, Accession Number 30409). A single colony from the fresh agar slants was transferred to a 500-mL Erlenmeyer flask containing 50 mL of seed medium that was composed of 20 g/L dextrin, 15 g/L soybean cake powder, 5 g/L glucose, 2.5 g/L yeast powder, and 1 g/L CaCO_3_. The seed culture was incubated at 33 °C for 24 h with 180 rpm of the shaking speed. Subsequently, the culture was inoculated at a 10 % (v/v) ratio, respectively, into a 500-mL Erlenmeyer flask containing 50 mL of the fermentation medium (CK: 60 g/L glucose, 35 g/L soybean cake powder, 2.2 g/L Na_2_SO_4_, 0.08 g/L K_2_HPO_4_, 0.1 g/L FeSO_4_·7H_2_O, 0.7 g/L Al_2_(SO_4_)_3_·7H_2_O, 2.5 g/L CaCO_3_) for control group, and fermentation medium supplemented additionally with 5 % mixed fatty acids (1.4 % oleic acid, 2.8 % linoleic acid, 0.3 % stearic acid, and 0.5 % palmitic acid) as the FAA group, followed by fermentation for 12 days.

Scale-up fermentation was performed in a 5-L fermenter. Cultures of mature flasks were transferred to the fermenter (Bailun Biotechnology Co., Ltd., China) containing 3 L of the medium (CK/FA group), supplemented with 0.2 % (v/v) silicone antifoam. Temperature, pH, and dissolved oxygen (DO) were controlled at 33 °C, 6.6, and 30 %, respectively. Initial agitation was set to 400 rpm and adjusted to a maximum 700 rpm based on DO levels. Aeration was initially provided at 3 L/min and increased to a maximum of 6 L/min depending on DO levels. Fermentation proceeded for 10 days, with ammonia added to maintain pH above 6.6, and sterile water used to maintain a constant volume of 3 L.

### 2.2 Measurement of strain incubation at physiological level

#### 2.2.1 Analysis of the fatty acid composition in cell membrane

Fatty acid methyl ester derivatization and subsequent gas chromatographic analysis was performed following the MIDI, USA, protocol for the identification of fatty acid methyl esters in the cell membrane [18]. The average carbon chain length was determined using Eq. (1).

(1) $$\text{Average}\;\text{carbon}\;\text{chain}\;\text{length}=\frac{\sum \left(\text{Fatty}\;\text{acid}\;\text{percentage}\times \text{Carbon}\;\text{number}\;\text{of}\;\text{fatty}\;\text{acids}\right)}{100}$$

Additionally, the flow index (U/S) of membrane fatty acids, which is the ratio of unsaturated to saturated fatty acids, was calculated based on Eq. (2).

(2) $$\text{Flow}\;\text{index}=\frac{\text{Percentage}\;\text{of}\;\text{unsaturated}\;\text{fatty}\;\text{acids}}{\text{Percentage}\;\text{of}\;\text{saturated}\;\text{fatty}\;\text{acids}}$$

#### 2.2.2 Cofactor measurement

Cells were rapidly quenched in an ice bath with 0.5 M perchloric acid (HClO_4_), followed by immediate cell disruption with an ultrasonic cell breaker (JY92-II, Xinzhi, China) under ice-bath condition [19]. Sonication parameters included a power setting of 325 W, with a pulse duration of 3 s on and 1 s off, totaling 10 min of processing time. After the disruption, cell debris was removed by centrifugation at 12,000 g for 10 min, and the supernatant was collected and filtered through a 0.22-μm membrane for subsequent analysis. Cofactor concentrations were determined using high-performance liquid chromatography (HPLC) on Waters e2695-2998 PDA system, equipped with a C18 column and a UV detection system set at 254 nm. The mobile phase consisted of a mixture of 5 % acetonitrile, 95 % sodium phosphate buffer (pH 7.0, 0.2 M), and 10 mM tetrabutylammonium bromide, and elution was performed at 25 °C with a flow rate of 1.0 mL/min.

#### 2.2.3 Cell viability measurement

Cell viability was assessed using the triphenyltetrazolium chloride assay [20]. The procedure involved mixing 200 μL of fermentation broth with 1 mL of sodium phosphate buffer (pH 7.0, 0.1 M) and 50 μL of a 0.5 g/L triphenyltetrazolium chloride assay solution. The mixture was incubated at 40 °C for 3 h. After the incubation, the samples were centrifuged at 12,000 g for 5 min to pellet the cells, followed by aspiration of the supernatant. Then, 1 mL of methanol was added to the cell pellet, and the mixture was subjected to ultrasonication for 30 min. Subsequently, the samples were centrifuged again at 12,000 g for 5 min. The absorbance of the resulting supernatant was measured at 485 nm using a spectrophotometer (SpectraMax 190, MD, USA).

#### 2.2.4 Enzyme viability measurement

Cell resuspension was carried out using a 100 mM Tris-HCl buffer (pH 7.5) supplemented with 20 % glycerol and 1 mM dithiothreitol. The suspension was subjected to ultrasonic disruption under ice-bath conditions with an ultrasonic cell breaker. Sonication parameters were set at 260 W power, with a 2-s pulse on and a 2-s pulse off, for a total duration of 30 min. After the ultrasonic disruption, the mixture was centrifuged at 12,000 g for 20 min to pellet debris, and the supernatant was collected for subsequent enzyme assays. Glucose 6-phosphate dehydrogenase (Zwf) activity was measured spectrophotometrically by monitoring the increase in absorbance of nicotinamide adenine dinucleotide phosphate (NADPH) at 340 nm at 30 °C [21]. Similarly, pyruvate kinase (Pyk) activity was determined by the decrease in absorbance of nicotinamide adenine dinucleotide (NADH) at 340 nm at 30 °C [22]. Citrate synthase (Cs) activity was assessed by the increase in absorbance of citryl-CoA at 412 nm at 30 °C [23], and pyruvate carboxylase (Pyc) activity was quantified by the decrease in NADH absorbance at 340 nm at 30 °C [24]. Enzyme activity was defined as a change of 0.01 in absorbance per minute, calculated using Eq. (3).

(3) $$U=\frac{\Delta A}{0.01\times t}\times D$$

U: enzyme activity, *ΔA*: the change in absorbance value during the reaction time, *t*: reaction time (min), *D*: dilution factor.

### 2.3 Transcript analysis of *S. cinnamonensis* induced by fatty acids

The strain was initially cultured in seed medium and then transferred to both CK and FA media for concurrent maturation and cultivation. Biomass was collected at the peak of monensin synthesis (144 h), followed by centrifugation at 12,000 g at 4 °C. The samples were promptly frozen in liquid nitrogen and stored at −80 °C pending RNA sequencing. Each group included three biological replicates. The samples were submitted to Genedenovo Biotechnology Co. Ltd., (Guangzhou, China) for transcriptome sequencing. RNA-Seq data were deposited in the National Center for Biotechnology Information database under the accession number PRJNA 1171199. Analysis of the RNA-Seq data was performed using EdgeR and DESeq2 software through an online platform (http://www.omicshare.com), adhering to standard protocols. Differentially expressed genes (DEGs) were identified with a threshold of |log_2_ fold change (fc)| ≥ 1 and a false discovery rate (FDR) < 0.05.

### 2.4 Real-time quantitative polymerase chain reaction (RT-qPCR) analysis

To validate the RNA-Seq findings, seven genes were selected for quantitative expression analysis using RT-qPCR [25]. Complementary DNA (cDNA) was synthesized from total RNA using the HiScript® QRT SuperMix for qPCR (+gDNA wiper) (Vazyme, China). The RT-qPCR reactions were performed with a ChamQ SYBR qPCR Master Mix (Vazyme, China) on a QuantStudio™ 5 Real-Time PCR System (Applied Biosystems, Foster City, CA, USA) following the manufacturer's protocol and with primers listed in Table S1. For normalization of the relative expression levels of the target genes, 16S rRNA was used as an endogenous control. The relative transcriptional levels were determined employing the 2^−ΔΔCT^ method [26], and the gene expression data were logarithmically transformed (base 2) for statistical analysis and validation.

### 2.5 Quantification of metabolites and packed mycelium volume (PMV)

Monensin and PMV were determined according to the established methods [9,27]. For the preparation of the crude extract, 1 g of fermentation broth was mixed with 50 mL of methanol and thoroughly blended. The mixture was then subjected to sonication for 30 min. Following this, the crude extract was filtered through a 0.45-μm organic filter membrane and transferred into liquid-phase vials. HPLC was employed for the determination, utilizing a C18 column (250 × 4.6 mm, 5 μm) and a mobile phase consisting of methanol, water, and glacial acetic acid in a ratio of 94:6:0.1. The flow rate was set at 0.7 mL/min. The detection wavelength was established at 520 nm, and the sample injection volume was 20 μL. The temperature of derivation was maintained at 98 °C. The diluent consisted of a 90:10 mixture of methanol and water. The derivatization reagent was prepared by dissolving 3 g of vanillin in a 95:2 mixture of methanol and sulfuric acid.

Triacylglycerols (TAGs) were extracted and purified with minor modification according to the previous method [28]. Briefly, cells were collected by centrifugation at 4 °C, 12,000 g for 1 min. A defined volume (4.5 mL) of chloroform: methanol (2:1) was added to 10 mg of lyophilized *S. cinnamonensis* mycelium and extracted by sonication in a water bath at 40 °C for 2 h, vertexing for 30 s every 30 min, followed by centrifugation (1000 g, 10 min) to obtain phase separation. The lower organic phase was collected, and the upper aqueous phase was added to 2 mL mixture of chloroform: methanol (85:15) for a second extraction. The two organic phases were put together and evaporated under a stream of nitrogen at room temperature. After redissolving, the samples were analyzed for TAGs and fatty acids (FAs) by thin-layer chromatography on silica gel. Following visualization with iodine staining, the TAGs and FAs bands were quantified using a gel imaging system.

### 2.6 Analysis of mycelium morphology

Fresh fermentation broth was centrifuged, and 2 g cells were rapidly fixed in a 1.5 mL centrifuge tube containing 2.5 % glutaraldehyde and stored at 4 °C for 24 h. After fixation, the fixative was removed, and the samples were rinsed three times with phosphate-buffered saline (PBS) buffer (pH 7.0) each. Subsequently, the samples underwent a concentration gradient ethanol elution process. Dehydrated samples were then placed in a critical point dryer for desiccation. The dried samples were mounted on a sample stage using conductive carbon adhesive, and a 120-s platinum (Pt) sputter coating was applied using an ion sputter coater. The samples were examined using a scanning electron microscope (SEM) (SU8010, Hitachi, Japan), and representative images were captured [29].

### 2.7 Statistical analysis

Group comparisons were conducted using one-way or two-way analysis of variance. Error bars denote the mean ± standard error of the mean. All statistical analyses and graphical representations were performed using GraphPad Prism version 10 (GraphPad Software, San Diego, USA). Statistical significance was determined at the following thresholds: ∗p < 0.05, ∗∗p < 0.01, ∗∗∗p < 0.001, and ∗∗∗∗p < 0.0001.

## 3 Results and discussion

### 3.1 Phenotypic assay on microbial monensin fermentation with FAA strategy

In our earlier study, four fatty acids, oleic acid, linoleic acid, stearic acid, and palmitic acid, predominantly consisting of the vegetable oil, were observed to have a positive role on the monensin fermentation, contributing to a significant increase of monensin titer by 8, 6, 2 and 3 folds, though inferior to the soybean oil (10 folds) (Fig. S1). With optimization of mixed fatty acids (1.38 % oleic acid, 2.78 % linoleic acid, 0.31 % stearic acid, and 0.50 % palmitic acid), the FAA rendered the strain a boosted monensin output high up to 14.8 g/L, which is 21.3 % higher than that of the soybean oil alone. Further with the FAA in a fed-batch fermentation at a 10-L level, the strain produced an industrially attractive level of 26.7 g/L, above 10 % higher than that of the common soybean oil. Thus, it was inferred that the suitable combination of mixed fatty acids for alternative to the vegetable oil was favorable for the microbial growth and monensin synthesis, which would be reported elsewhere.

**Fig. 1:**
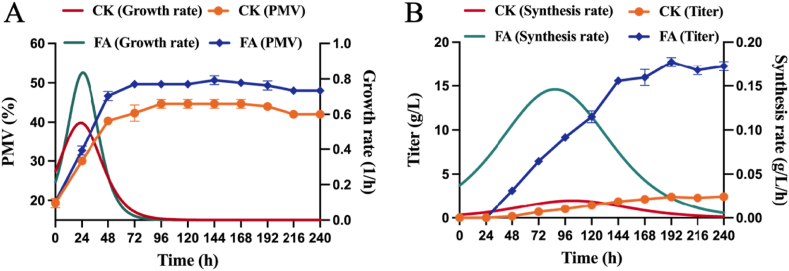
Effect of the FAA strategy on fermentation parameters of *S. cinnamonensis*. A, Packed mycelium volume (PMV) and growth rate; B, Monensin titer and synthesis rate. **CK**: control, **FA**: fatty acids group.

This experiment was purposed to evaluate the effect of FAA strategy on the microbial growth and monensin synthesis at a 5 L fermenter level. As given in Fig. 1A, the cell concentration of *S. cinnamonensis* exhibited a progressive increase throughout the growth phase, resulting in an initial increase and then decline of the growth rate. At 96 h, the fermentation entered a stabilization phase, which was marked by diminished growth and slowdown in the growth rate. Remarkably, the cell concentration and specific growth rate with the FAA surpassed that of the control group at all times. The former cell concentration reached 51 % at 120 h, which is 13 % higher than the latter's. This difference highlights the significant enhancement in *S. cinnamonensis* proliferation due to the FAA. Under the FAA strategy, the synthesis rate of monensin was significantly high, and the monensin titer maximized to be 17.72 g/L at 192 h, 7.36 times that of the control group (Fig. 1B). These results evidenced the substantial impact of FAA on monensin biosynthesis. Interestingly, despite the intervention of fatty acids, the fermentation kinetics of monensin remained in a growth-partial coupling mode, while the peak of product synthesis was advanced by 24 h. This suggested a realignment of carbon fluxes from cell growth to product biosynthesis, offering insights into the mechanism by which the FAA strategy enhanced monensin production.

### 3.2 Effect of fatty acids on the physiological level of monensin-producing strain

FAA strategy can markedly enhance the fermentation of *S. cinnamonensis*, which holds a substantial significance in the realm of industrial microbial fermentation. Considering that the precise mechanism by which FAA augments monensin synthesis remains unclear, it becomes very crucial to understand how *S. cinnamonensis* responds to the FAA and efficiently synthesizes monensin at a physiological level. Therefore, physiological parameters of cells, i.e., cellular resistance, cell membrane fluidity, key enzyme activity, cell viability and intracellular energy cofactors, were investigated.

#### 3.2.1 Cellular resistance

**Fig. 2:**
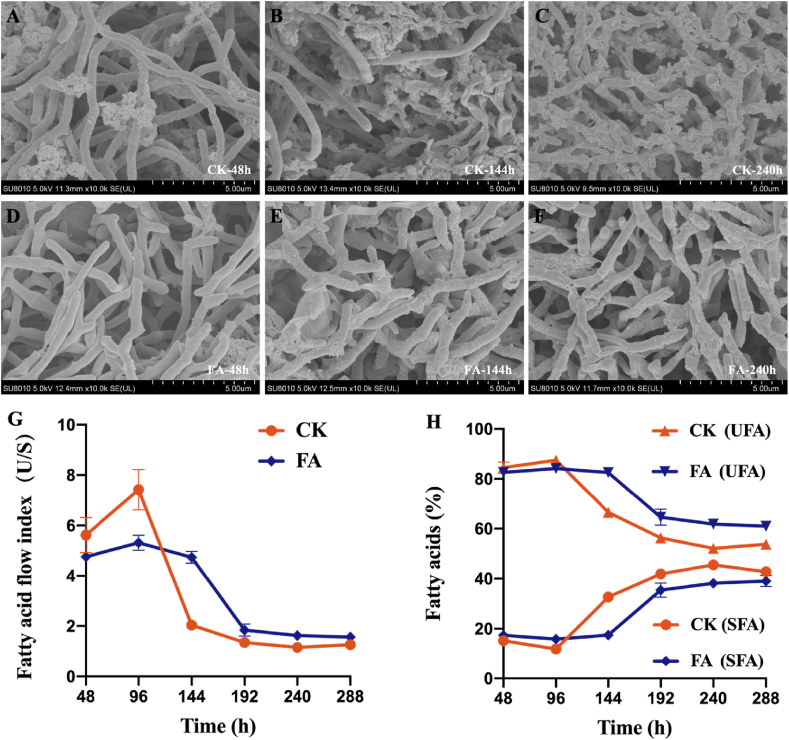
Effect of the FAA on cell morphology and cell membrane during *S. cinnamonensis* fermentation. A–F, cell morphology; G, total unsaturated fatty acids and saturated fatty acids; UFA: unsaturated fatty acids, SFA: saturated fatty acids; H, Fatty acid flow index. **CK**: control; **FA**: fatty acids group.

In industrial production process, cellular resistance is strongly related to cell morphology [30]. Previous research has established a correlation between alterations in cell morphology and product synthesis in *Streptomyces* species [31]. As shown in Fig. 2, the mycelium in the CK remained consistently thin and long, with less monensin synthesis (Fig. 2A–C). The FA also had thin and long mycelium in the early stages (Fig. 2D), like the control group. However, in the middle and later stages of fermentation, the mycelium gradually became short and thick (Fig. 2E and F), and the monensin titer increased rapidly (Fig. 1B). Therefore, it can be inferred that the short, thick, rod-like mycelial state was conducive to the biosynthesis of secondary metabolites. It is important to note that in the early stage of fermentation, *S. cinnamonensis* extensively utilized glucose, and the mycelium in both groups was in the primary metabolism stage, presenting a thin filamentous state. As glucose was depleted, *S. cinnamonensis* began to utilize fatty acids, and the mycelial biomass gradually transformed into a short, thick, rod-like morphology, which was helpful to the monensin synthesis. The control group that had no FFA did not show significant changes in mycelial state. Accordingly, the above result indicates that the FAA indeed induced the transformation of filamentous morphology. This result is in line with the literature that a short filamentous state of some *Streptomyces* species is favorable to the product synthesis, whereas maintaining a long filamentous morphology can adversely affect the rate of product synthesis [30,32]. Consequently, the FAA strategy altered the cellular morphology, thus enhancing the cellular resistance as well as the synthesis of secondary metabolites.

#### 3.2.2 Cell membrane fluidity

It is generally acknowledged that the alteration in fatty acid composition within cell membranes can influence the substrate transport [33,34]. Herein, we hypothesized that, the FAA also act on the cell membrane and modify the fatty acid composition of it towards easy substrate transport. To verify it, this experiment was made to analyze the cell membrane fluidity, with results depicted in Fig. 2 G. The types of fatty acids in the cell membranes of both groups were consistent. As the fermentation time progressed, there was an obvious increase in total saturated fatty acids and a corresponding decrease in unsaturated fatty acids within the cell membrane (Fig. 2G). Strikingly, *S. cinnamonensis* M6002 exhibited a slower increase of saturated fatty acids and reduction of unsaturated fatty acids with the FAA, as compared to the control group during the middle and late stages of fermentation. It is evident that the FAA tended to stabilize the composition of fatty acids in the cell membrane, contributing to a higher ratio of unsaturated fatty acids in the cell membrane. The flow index (U/S) of cell membrane fatty acids, a key determinant of cell membrane permeability [35], increased due to the FAA during the middle and late stages of fermentation (Fig. 2H). Given that unsaturated fatty acids confer greater flexibility than their saturated counterparts, it is inferred that the FAA should enhance the cell membrane fluidity, benefiting substance transport and energy metabolism. Consequently, this led to high efficiency in the translocation of nutrients such as fatty acids, glucose, and amino acids to the intracellular compartment, thereby potentially enhancing monensin production.

#### 3.2.3 Key enzyme activity

**Fig. 3:**
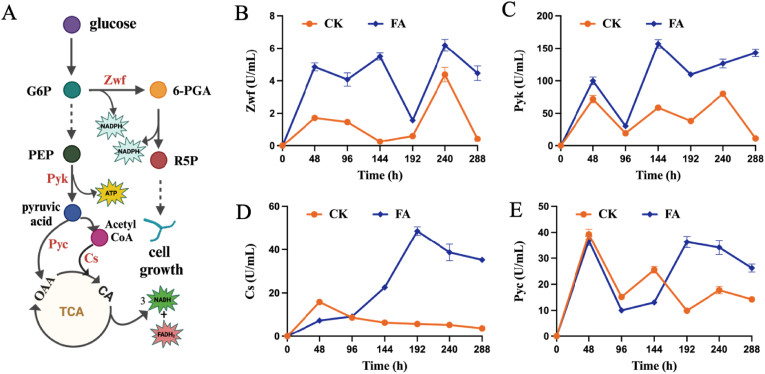
Effect of the FAA strategy on the key enzymes in the metabolic process of *S. cinnamonensis*. A, *S. cinnamonensis* primary metabolic pathways; key enzymes are indicated in red; G6P: glucose-6-phosphate; PEP: phosphoenolpyruvate; 6-PGA: 6-phosphogluconic acid; R5P: ribose 5-phosphate; OAA: oxaloacetic acid; CA: citric acid; B, glucose 6-phosphate dehydrogenase (Zwf); C, pyruvate kinase (Pyk); D, citrate synthase (Cs); E, pyruvate carboxylase (Pyc). **CK**: control; **FA**: fatty acids group.

To elucidate the impact of FAA on the metabolic pathways involved in the monensin biosynthesis, the activity of some key enzymes was analyzed (Fig. 3). These key enzymes include Zwf within pentose phosphate pathway (PPP), Pyk in Embden-Meyerhof-Parnas (EMP) pathway, and Cs and Pyc related to tricarboxylic acid (TCA) cycle (Fig. 3A). Zwf, an initial rate-limiting enzyme of the PPP, exhibited a biphasic activity pattern, initially increasing and then decreasing. Outstandingly, the enzyme activity of Zwf presented a relatively high tendency with the FAA throughout the fermentation process. The result indicated that the FAA boosted the metabolic flux through the PPP by elevating the activity of this key enzyme, consequently promoting the production of pentose and NADPH (Fig. 3B). This finding is aligned with the observed result in Fig. 1A.

Pyk, another key enzyme that catalyzes the conversion of phosphoenolpyruvate and ADP to pyruvate and ATP, is recognized as a key rate-limiting step in the glycolysis. The activity of this enzyme is intrinsically linked to the rate at which these metabolic intermediates are channeled into the TCA cycle [36]. As depicted in Fig. 3C, the activity of Pyk in the control group exhibited minor fluctuations throughout the fermentation process. In contrast, the FAA displayed a steady upward trend, surpassing the control group's activity level at all stages. Particularly, at 144-h of the fermentation, the Pyk activity doubled that of the control group. This relative increase of Pyk activity during the monensin synthesis phase was helpful for the high ATP production, which is likely consistent to the strong energy demand at the fermentation phase of secondary products.

Cs catalyzes the condensation of acetyl-CoA with oxaloacetate to form citric acid, representing the initial and rate-limiting step of the TCA cycle [37]. As shown in Fig. 3D, the activity of Cs in the control group exceeded that of the FAA before 96 h, after which it progressively declined. Conversely, the activity of Cs with the FAA increased gradually post-96 h, peaking at 192 h with a value of 48.57 U/mL, over eight folds higher than that of the control group. Similarly, after 144 h of the fermentation, Pyc with the FAA showed a strong enzyme activity. Collectively, these findings suggest that the FAA strategy facilitate the TCA metabolism, thereby releasing additional energy to support the bacterial growth and product synthesis.

#### 3.2.4 Cell viability and intracellular energy cofactors

**Fig. 4:**
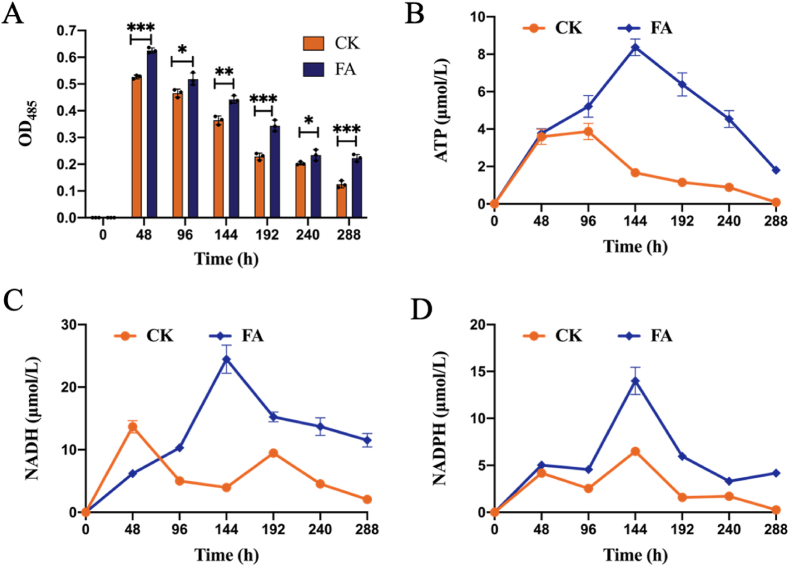
Effect of the FAA strategy on cell viability and energy cofactors in the metabolic process of *S. cinnamonensis*. A, cell viability; B, ATP; C: NADP; D: NADPH. **CK**: control; **FA**: fatty acids group. ∗p < 0.5, ∗∗p < 0.01, ∗∗∗p < 0.001, ∗∗∗p < 0.0001.

The monensin biosynthesis by *S. cinnamonensis* is an energetically demanding process, with intracellular energy levels being closely correlated with the cellular vitality [28]. To elucidate the metabolic impact of FAA strategy on *S. cinnamonensis*, we assessed the cellular viability, as shown in Fig. 4. After 48 h of the fermentation, cell viability showed a gradual decrease in both fermentation processes. Meantime, the FAA resulted in a higher cellular viability throughout the fermentation process compared to the control group. At the end of fermentation, the viability of control group had plummeted by 76.3 % relative to the 48-h mark, whereas the FAA exhibited a less decrease of 64.3 %. This suggested the FAA strategy was indeed effective in preserving the cellular viability. Presumably, the relatively high cellular viability should supply a high ATP yield to fuel both bacterial proliferation and the biosynthesis of secondary metabolites, so intracellular energy cofactors, including ATP, NADP, and NADPH, were detected (Fig. 4B–D).

The intracellular concentrations of ATP, NADH, and NADPH initially ascended and basically declined for both of the fermentation process. Comparative analysis at equivalent time points revealed that the FAA exhibited a significantly higher level of these energy cofactors compared to the control group. Particularly, the energy cofactor concentration peaked at 144 h. Integrating the data from Fig. 4, it is evident that the increased enzyme activity facilitated the rapid accumulation of intracellular cofactors, thereby supplying the necessary energy for the monensin synthesis. Consequently, an elevated intracellular cofactor content significantly promoted the synthesis of monensin.

### 3.3 Transcriptomic insights into fatty acid-induced monensin production

**Fig. 5:**
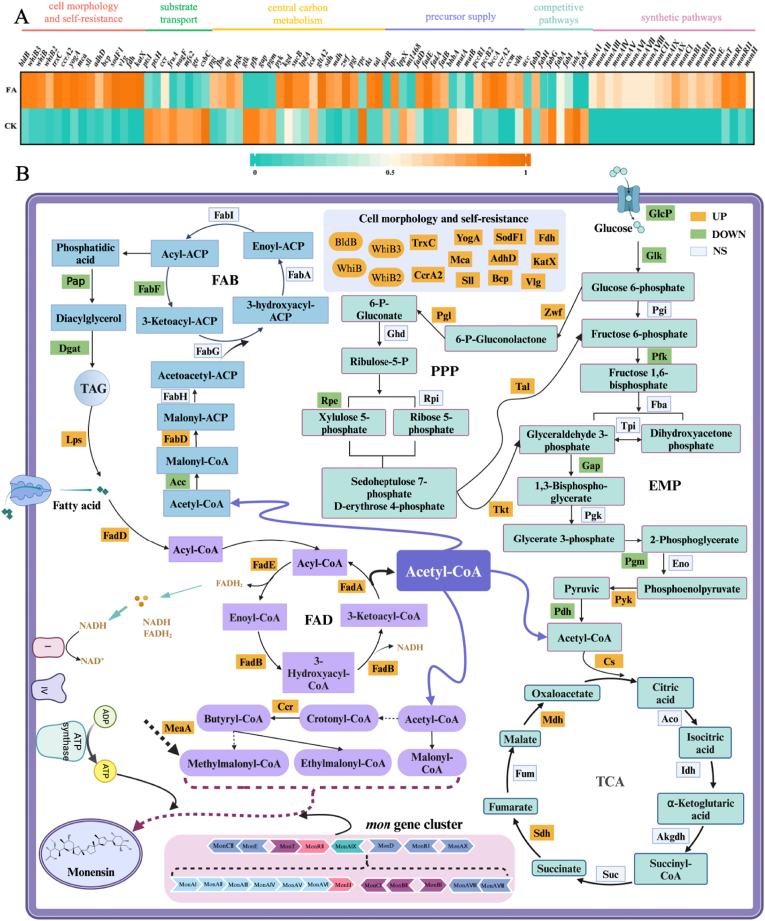
Carbon redirection analysis of fatty acids promoting monensin super-high yield. A, Key differential gene; B, Carbon redirection analysis; gray: cell morphology and self-resistance; cell membrane: substrate transport; green: central carbon metabolism; purple: precursor supply; blue: competitive pathway; pink: synthetic pathway.

The above results show that mixed fatty acids significantly enhanced the monensin production in *S. cinnamonensis* by intensifying the carbon flow. However, the molecular basis for this redirection of carbon flux towards product synthesis remains unclear. To address this, a comprehensive transcriptomics analysis was conducted to uncover the underlying molecular mechanism. In Fig. S2, a total of 4157 DEGs were identified with the FAA relative to the control group, including 778 up-regulated and 3379 down-regulated genes. Among them, genes associated with the cell morphology and self-resistance exhibited significant differences. Pathway enrichment analysis of the DEGs reveals that the most significant categories to Metabolism, Environmental Information Processing, and Cellular Processes were enriched with 652, 177, and 96 genes, respectively (Fig. S3). Genes related to the cell growth and product synthesis were particularly enriched. The gene expression trend observed by RT-qPCR was consistent with those from the transcriptome data, validating the reliability of the RNA-Seq analysis in this study (Fig. S4). In summary, these metabolic pathway alterations suggest that the FAA realigned the allocation of carbon sources within cells, and effectively redirected the carbon flux. By integrating these findings with KEGG pathway enrichment analysis and GO functional annotation, we systematically investigated six pivotal modules, involving the cell morphology and self-resistance, substrate transport, central carbon metabolism, precursor supply, competitive pathways, and synthetic pathways (Fig. 5).

#### 3.3.1 Cell morphology and self-resistance

Within the Biological Regulation (GO: 0065007) category, a significant upregulation of genes associated with the morphological differentiation of *Streptomyces* species was observed. These genes included *bldB*, *whiB3*, *whiB*, and *whiB2*. Existing literature corroborates our physiological experiments, indicating that the morphology of *Streptomyces* species significantly influenced the production of secondary metabolites [31]. Findings align with this, as an increase in monensin production in *S. cinnamonensis* corresponds to morphological changes. Additionally, among the 45 genes related to the cellular surface (GO: 0098754), 15 genes exhibited an upregulation with the FAA, which included 4 genes in the monensin biosynthesis cluster, *monAV*, *monAIV*, *monAIII*, and *monAII*, as well as 11 genes (*trxC*, *ccrA2*, *yogA*, *mca*, *sll0086*, *adhD*, *bcp*, *sodF1*, *v1g238856*, *fdh* and *katX*) related to the oxidative stress response (Fig. 5A). These results suggest that the FAA indeed modified the cell morphology and enhanced cellular metabolism in *S. cinnamonensis*, thereby contributing to the ultra-high monensin yield.

#### 3.3.2 Substrate transport

Two sugar transport systems, phosphotransferase system and major facilitator superfamily, can play an important role in the substrate transport for monensin synthesis [38]. In this experiment, transcriptomic analysis revealed that the expressions of key phosphotransferase system genes, including *ptsI* (encoding EI), *ptsH* (encoding Hpr), *ccr* (encoding EIIA), *fruA* (encoding EIIC), and *nagF* (encoding a glucose/sucrose transporter subunit), were downregulated under the FAA. And major facilitator superfamily, known to be involved in sugar transport in *Streptomyces*, exhibited a similar trend in gene expression, with *mfs2*, *gtr*, and *csbC* genes also showing the downregulation (Fig. 5A). These findings suggest that the sugar uptake was diminished during the peak period of cellular antibiotic synthesis, redirecting energy flow towards the synthesis of target products with the FAA strategy. Additionally, previous studies have indicated that the presence of glucose in the growth medium enhances fatty acid oxidation by elevating the uptake rate of fatty acids and the activity of acyl-CoA synthetase [39]. Consequently, it can be assumed that, during the early phase of fermentation with the FAA, glucose facilitated the cellular influx of fatty acids and the subsequent synthesis of lipids for energy storage. In the later stages of fermentation, when glucose became depleted, lipids were very likely catabolized to sustain essential cellular functions and support product synthesis [39].

#### 3.3.3 Central carbon metabolism

Central carbon metabolism (CCM), encompassing EMP, TCA, and PPP, is fundamental to an organism's ability to maintain the normal growth and development. This metabolic process supplies cells with energy and provides precursors and cofactors for various metabolic pathways [40]. Accordingly, the CCM was also taken into consideration with transcriptomics analysis (Fig. 5). As depicted in Fig. 5A, the expression of key glycolytic genes related to EMP pathway, including *pgi* (encoding phosphoglucose isomerase), *fba* (encoding fructose-bisphosphate aldolase), *tpi* (encoding triosephosphate isomerase), and *pgk* (encoding phosphoglycerate kinase), remained stable. In contrast, the expression of genes *glk* (encoding glucokinase), *pfk* (encoding phosphofructokinase), *gap* (encoding tglyceraldehyde-3-phosphate dehydrogenase), and *pgm* (encoding phosphoglycerate mutase) exhibited a downward trend. Meanwhile, the expression of *pyk* that encodes pyruvate kinase, was upregulated, which is in accordance with the physiological responses observed at the cellular level (Fig. 3C). These findings suggest that the FAA attenuated the metabolic activity of EMP pathway. In the TCA cycle (Fig. 5A), expression levels of the genes *kgd*, *sucB*, and *lpdA*, encoding α-ketoglutarate dehydrogenase, and *icd* encoding isocitrate dehydrogenase, did not change significantly, which may be attributed to the suppression of their expression due to the abundance of ATP and NADH generated by fatty acid oxidation [41]. Nevertheless, the expression of *gltA2* (encoding citrate synthase), *sdh* (encoding succinate dehydrogenase), and *mdh* (encoding malate dehydrogenase) was notably upregulated (Fig. 5A). The activation of these three genes suggests a slight enhancement of the overall TCA cycle strength under the FAA. In addition, our analysis identified several upregulated genes associated with the PPP, they were *zwf* (encoding glucose-6-phosphate dehydrogenase), *pgl* (encoding 6-phosphogluconolactonase), *tkt* (encoding transketolase), and *tal* (encoding transaldolase) (Fig. 5). Briefly, the FAA strategy modulating the CCM pathway not only provided additional reducing power for the antibiotic production but also sustained the robust vitality of cells [38], which is consistent with our above results on the phenotypic type and physiological state of monensin-producing strain.

#### 3.3.4 Precursor supply

**Fig. 6:**
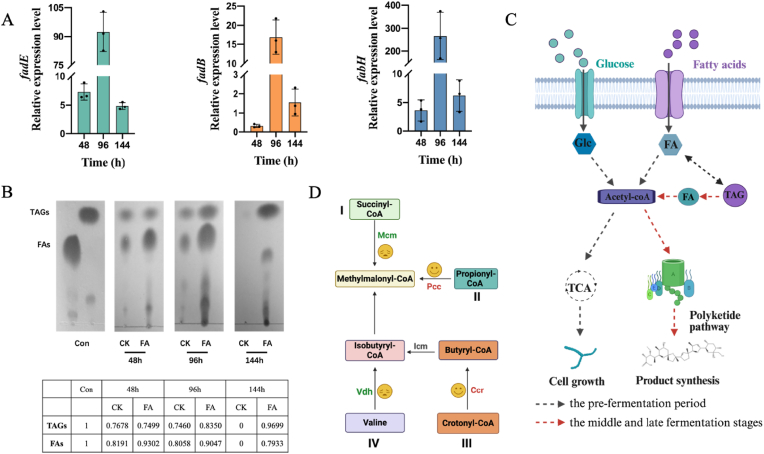
Analysis of precursor supply for monensin synthesis under FAA strategy. A, qPCR of key genes; B, thin-layer chromatography results of FAs and the TAGs; Tabular data are expressed as optical density; C, carbon source division of labor strategy; D, synthesis pathway of methylmalonyl-CoA.

Monensin biosynthesis requires a substantial supply of precursors, including malonyl-CoA, ethylmalonyl-CoA, and methylmalonyl-CoA [42,43]. It is extremely crucial to maintain reasonable precursor concentrations for the efficient product synthesis [44]. Therefore, it is necessary to concentrate on the change of the acetyl-CoA metabolic pathway that is responsible for the synthesis of these precursors under the FAA strategy (Fig. 6). During the initial phase of microbial growth and development, the expression of key genes in fatty acid degradation (*fadE*, *fadB*) and fatty acid synthesis (*fabH*) exhibited a biphasic pattern, initially increasing and then decreasing (Fig. 6A), which is consistent with the concentration change of fatty acids (Fig. 6B). Notably, the TAG content continued to increase at the initial phase (Fig. 6B), suggesting that *S. cinnamonensis* converted fatty acids to TAG for energy storage via acetyl-CoA to ensure a suitable concentration of precursors. As anticipated, the transcriptomic data revealed a significant upregulation of genes associated with TAG degradation (i.e., *lps*), while genes related to TAG synthesis, including *lppX* and *mt1468*, were downregulated in the middle and late stages of fermentation (Fig. 6A and B). Together with the up-regulated expression levels of fatty acid degradation genes, *fadE*, *fadB,* and *fadA* in *S. cinnamonensis* (Fig. 5B), we argue that the TAG stored previously at initial phase of microbial growth began to degrade and provided more acetyl-CoA for the monensin synthesis in the middle and late fermentation stage. This argument is completely consistent with the recent literature uncovering that TAGs accumulate at initial fermentation time and are degraded at middle-late stage [28]. And this process can redirect the carbon flux from both intracellular TAGs and extracellular substrates into the polyketide biosynthesis. To date, this interesting phenomenon was found for the first time in *S. cinnamonensis* after other *Streptomyces* strains, i.e., *S. coelicolor*, *S. venezuelae*, *S. rimosus* and *S. avermitilis* were reported by Wang et al. [28]. In summary, it can be speculated that, at the initial phase, microbial cells should synthesize a substantial amount of acetyl-CoA and store as an energy molecule in TAG. And at the middle and late stages of antibiotic synthesis, these acetyl-CoA precursors should be predominantly supplied through the TAG hydrolysis and fatty acid β-oxidation, thus redirecting the acetyl-CoA flux toward the polyketide synthesis (Fig. 6C).

In addition to the acetyl-CoA, the synthesis of other precursors is essential and involves various biochemical pathways. Malonyl-CoA and ethylmalonyl-CoA are generated through the carboxylation of acetyl-CoA and butyryl-CoA, respectively, and in contrast, the production of methylmalonyl-CoA is much more essential, which follows an intricate biosynthetic route [45]. Up to date, four pathways are widely acknowledged to contribute to formation of the methylmalonyl-CoA (Fig. 6D). Methylmalonyl-CoA is formed from succinyl-CoA through methylmalonyl-CoA mutase (Mcm) isomerization. Our transcriptomic analysis revealed that genes encoding Mcm (*bhbA*, *mutA*, *mutB*) were downregulated, suggesting this was not a primary route for methylmalonyl-CoA synthesis. Genes (*pccB1*, *pccB2*, *bccA*) relating to propionyl-CoA carboxylase that catalyzes the production of methylmalonyl-CoA from propionyl-CoA were upregulated (Fig. 5A), implying the involvement of this pathway in methylmalonyl-CoA generation. Moreover, the methylmalonyl-CoA is supplied through a metabolic pathway that includes crotonyl-CoA reductase (Ccr) when *S. cinnamonensis* is cultured in an oil-contained medium [46]. Although the precise reaction of this complex pathway remains obscure, our transcriptomic data highlighted that a significant upregulation of the *ccrA2* gene encoding Ccr increased expression of the MeaA protein-coding gene (*ecm*), located at a 36 base-pair downstream of the Ccr gene. Additionally, the absence of high expression in valine dehydrogenase gene (*vdh*) suggested it may not be a significant contributor, though valine catabolism is another potential pathway. Consequently, we hypothesize that the biosynthesis of methylmalonyl-CoA for monensin production should be primarily facilitated with using the propionyl-CoA carboxylase and protein complex encoded by *ccrA2* and *ecm*, respectively (Fig. 6D).

#### 3.3.5 Competitive pathway

The literature have demonstrated that *de novo* fatty acid synthesis is facilitated through a type II fatty acid synthase system in *Streptomyces* species [47]. And the synthesis involves repeated decarboxylation condensation reactions between acyl-CoA activated substrates, which parallels the biosynthesis of polyketides, so a vibrant fatty acid synthesis is actually undesirable for the monensin biosynthesis as they share the common acetyl-CoA precursor. This experiment focused on the fatty acid synthase system with transcriptomic analysis (Fig. 5). As shown in Fig. 5A, five key genes (*acc*, *fabH*, *fabG*, *fabA*, and *fabI*) consisting of the system were downregulated obviously during peak antibiotic production, suggesting a diminished capacity for the fatty acid biosynthesis. This appealing result indicates the fatty acid biosynthesis pathway became weak under the condition of the rich acetyl-CoA synthesis induced by mixed fatty acids. Considering the subtle increase of the TCA cycle that serves as a conduit for acetyl-CoA within the central carbon metabolism module, it can be inferred that the acetyl-CoA flux should redirect towards the polyketide synthesis, thereby potentially augmenting antibiotic biosynthesis. Consequently, the subdued fatty acid synthesis led by FAA strategy was favorable to the monensin synthesis.

#### 3.3.6 Synthetic pathway

Currently, at least 20 genes within the monensin biosynthetic gene cluster (*mon*) have been identified to be related to the monensin biosynthesis. These genes have been categorized into polyketide synthase genes, modifier genes and pathway-specific regulatory genes, based on their roles in monensin synthesis [48]. The ensuing experiment also focused on them with transcriptomic analysis. As given in Fig. 5A, eight open reading frames encoding type I polyketide synthase multifunctional enzymes (*monAI* to *monAVIII*) were present in the cluster, and their expression was significantly upregulated with the FAA. In addition to the polyketide synthase genes, the expression of numerous modifier genes in the gene cluster (*monCII*, *monAIX*, *monAX*, *monCI*, *monBI*, *monBII*, *monD*, *monE*, and *monT*) was upregulated. Results indicate that these modifier genes should be correlated with the monensin biosynthesis in the *mon* gene cluster. Among them, *monCII*, *monAIX*, and *monAX* genes encoding thioesterases were crucial for terminating chain reactions and releasing product molecules [49,50]. The genes *monCI*, *monBI*, and *monBII* encode epoxide hydrolase/cyclases involved in the cyclization of polyketides [13,51]. And the genes *monD* and *monE* encoding the hydrolase and methylase, respectively, were responsible for the processing and post-modification of the product molecule at the final steps in monensin biosynthesis [52]. Additionally, the *monT* gene was predicted to encode an antibiotic transmembrane transporter protein, facilitating the extracellular export of monensin [7]. Finally, these putative pathway-specific regulatory genes, *monRI*, *monRII*, and *monH*, in the monensin biosynthesis were also upregulated with the FAA. This is very possibly owing to the fact that genes *monRI*, *monRII*, and *monH* act as SARP family positive regulators, TetR family negative regulators, and LAL family positive regulators, respectively [8,12,53]. Consequently, the FAA facilitated the shift of carbon flow to product synthesis from filamentous growth through the high expression of the monensin biosynthesis gene cluster in *S. cinnamonensis*, thereby increasing monensin titers.

### 3.4 Mechanism of ultra-high monensin productivity of strain with using the FAA strategy

The physiological and transcriptional response of *S. cinnamonensis* to the FAA strategy suggests that the direction of carbon flux was largely crucial for monensin synthesis (Fig. 5B). During the pre-fermentation phase, the nutrient uptake and metabolism were enhanced, thus accelerating the cell membrane fluidity. The presence of glucose facilitated rapidly the fatty acid uptake, directing the carbon primarily towards cell growth. Concurrently, fatty acids underwent an intracellular β-oxidation, generating acetyl-CoA that could feed into the polyketide pathway, and was also used for the synthesis and storage of TAGs. As the fermentation progressed and fatty acids were depleted, TAGs were mobilized, and β-oxidation was activated to sustain the supply of precursors and energy for synthesis. Concurrently, the PPP and TCA cycles, which provided the essential energy, were upregulated. In contrast, the EMP and lipid synthesis pathways were downregulated, which competed for the carbon resource. Ultimately, the high expression of the monensin synthetic gene cluster, induced by fatty acids, redirected the carbon flux from cellular biosynthesis to product synthesis, thus resulting in the ultra-high yield of monensin.

## 4 Conclusion

An FAA strategy effectively enhanced the monensin production from *S. cinnamonensis*. This was due to the fact that, physiologically, the FAA facilitated the cellular resilience, key enzyme activity, cellular vitality, and energy cofactor level, thus resulting in a substantial increase in carbon flow. Genetically, the FAA strategy significantly upregulated the expression of genes related to product synthesis, precursor provision, and energy metabolism, also redirecting the carbon source away from cellular biosynthesis at initial/middle stage towards the polyketide synthesis at late stage in strain. This regulatory shift amplified the expression of synthetic gene clusters, thereby achieving an unprecedented monensin yield. These findings not only established a foundation for the industrial-scale production of monensin but also paved the way for future research focus on genetic modification or metabolic engineering of industrial strains.

## CRediT authorship contribution statement

**Shanfei Zhang:** Writing – original draft, Methodology, Investigation, Funding acquisition, Data curation. **Qingming Hou:** Methodology, Investigation. **Zhenhua Wang:** Validation, Investigation. **Dandan Tian:** Writing – original draft, Investigation. **Xianyuan Zhang:** Writing – original draft, Investigation. **Yan Zhang:** Methodology, Data curation. **Qun Wu:** Writing – review & editing, Supervision. **Fubao Sun:** Writing – review & editing, Supervision, Funding acquisition.

## Declaration of competing interest

The authors declare the following financial interests/personal relationships which may be considered as potential competing interests: Shanfei Zhang, Qingming Hou, Zhenhua Wang, Dandan Tian, Xianyuan Zhang, Yan Zhang are currently employed by Shandong Shengli Bioengineering Co., Ltd.
