# Supplementary material for: Fatty acid addition strategy redirected the metabolic flux towards an ultra-high monensin productivity of Streptomyces cinnamonensis
Source: Synth Syst Biotechnol. 2025 Feb 18;10(2):532–42. doi: 10.1016/j.synbio.2025.02.009 (PMC11903796; doi:10.1016/j.synbio.2025.02.009)
Supplement: Multimedia component 1 [file mmc1.docx]

**Supporting Information**

***Fatty acid addition strategy redirected the metabolic flux towards an ultra-high monensin productivity of Streptomyces cinnamonensis***

**Content**

**Figures and Tables**

**Fig. S1** Effect of different fatty acids on the titer of monensin.

**Fig. S2** Differentially expressed genes and PCA in *Streptomyces cinnamonensis* M6002 under fatty acid addition. A: PCA; B: volcano map of differentially expressed genes.

**Fig. S3** GO and KEGG enrichment analysis of DEGs in *Streptomyces cinnamonensis* M6002 under fatty acid addition. A: GO primary classifications; B: GO secondary classifications; C: KEGG primary classifications; D: KEGG secondary classifications.

**Fig. S4** Correlation of RNA-seq (x axis) with qPCR data (y axis) using the log_2_ fold change measure of the genes differentially expressed across the two gene-expression platforms under correlation analysis.

**Table S1** The primers used in RT-qPCR analysis.

**Figures and Tables**


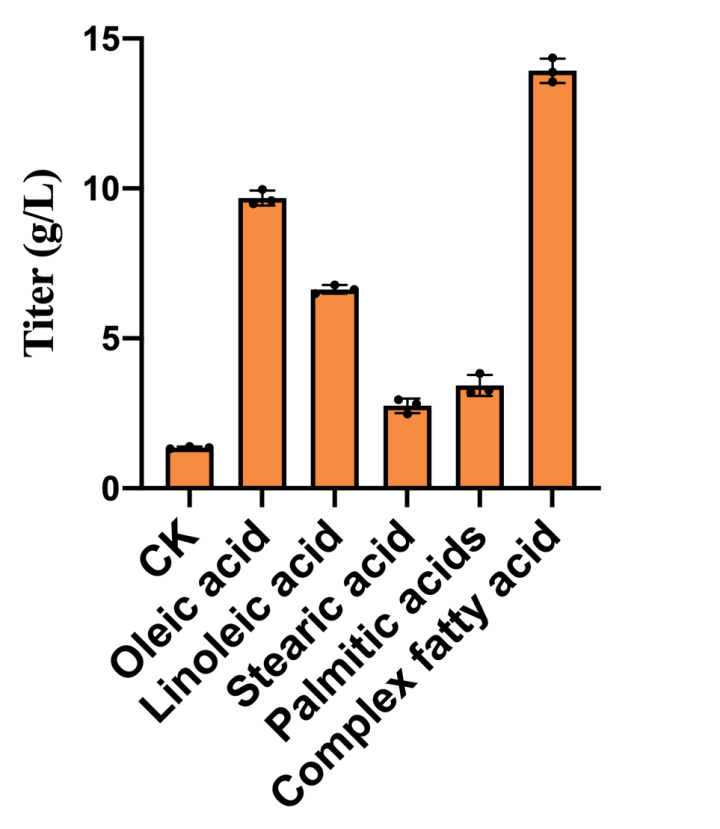


**Fig. S1** Effect of different fatty acids on the titer of monensin. CK: control check.


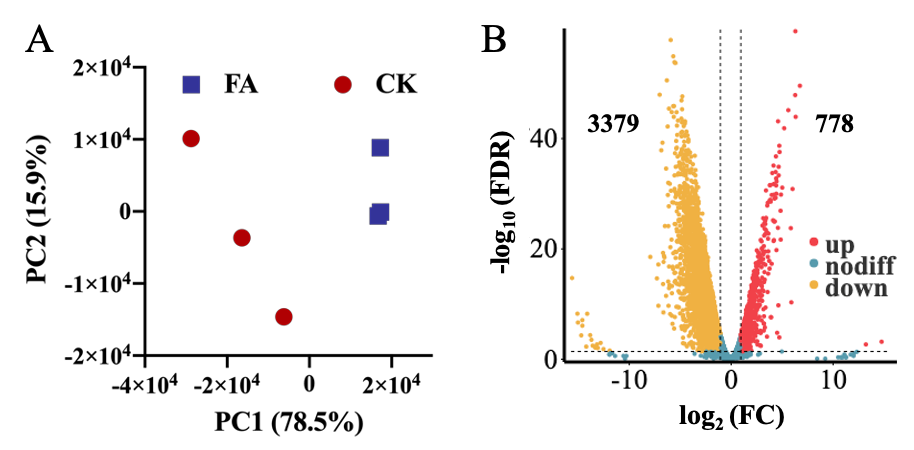


**Fig. S2** Differentially expressed genes and PCA in *Streptomyces cinnamonensis* M6002 under fatty acid addition. A: PCA; B: volcano map of differentially expressed genes.

**
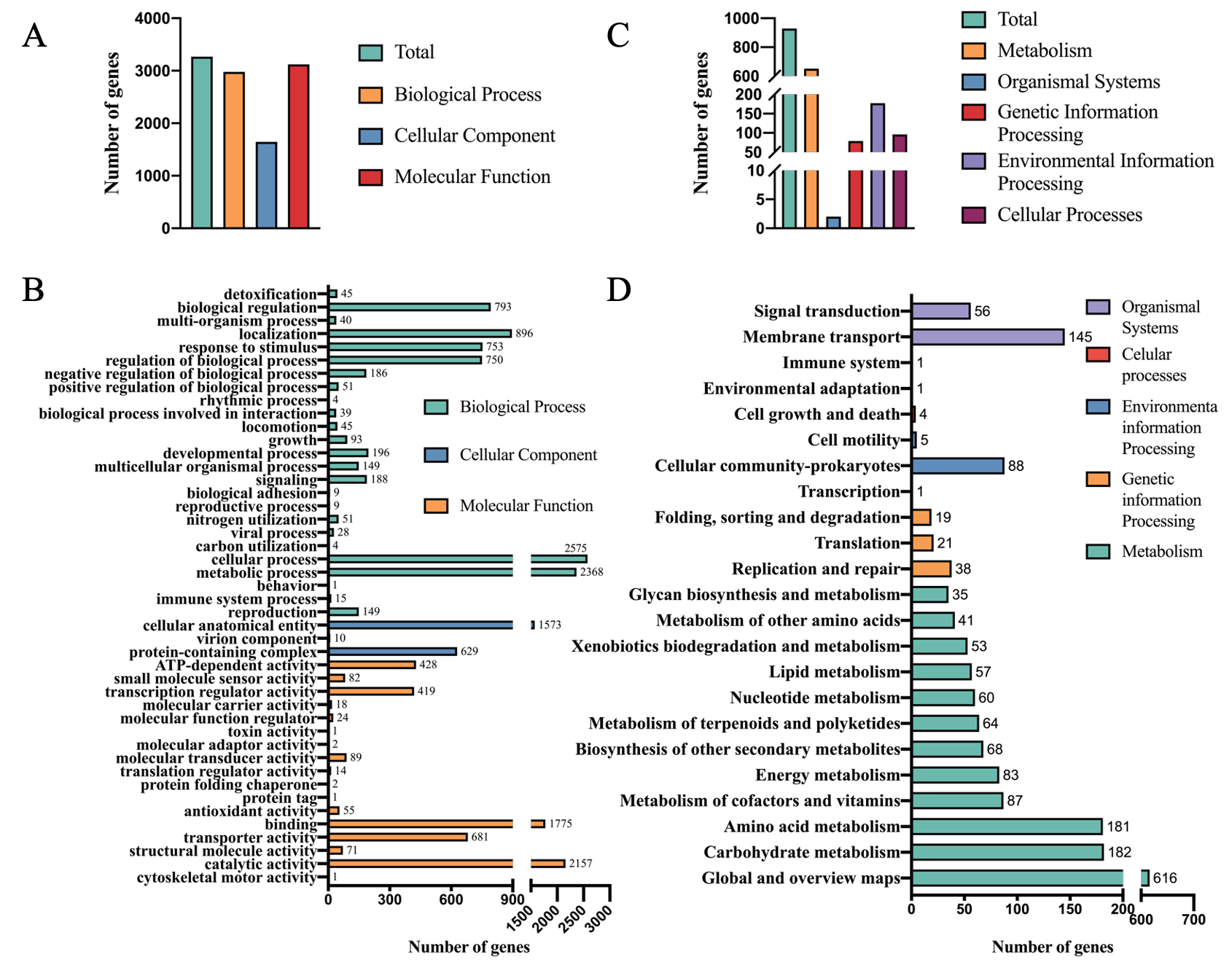
**

**Fig. S3** GO and KEGG enrichment analysis of DEGs in *Streptomyces cinnamonensis* M6002 under fatty acid addition. A: GO primary classifications; B: GO secondary classifications; C: KEGG primary classifications; D: KEGG secondary classifications.


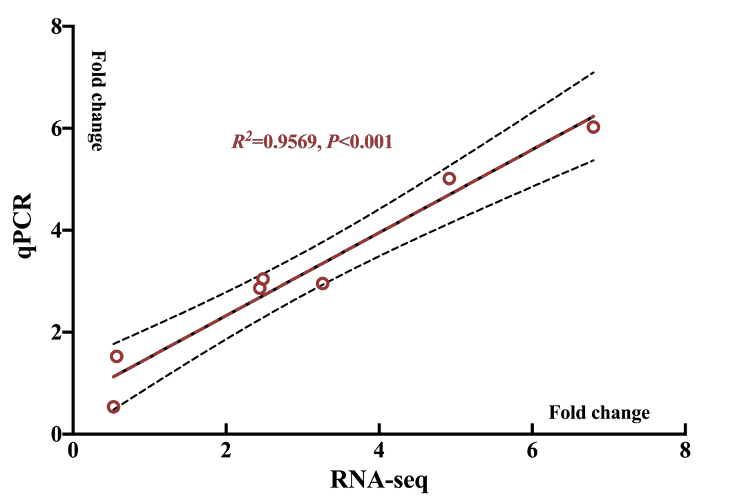


**Fig. S4** Correlation of RNA-seq (x axis) with qPCR data (y axis) using the log_2_ fold change measure of the genes differentially expressed across the two gene-expression platforms under correlation analysis.

**Table S1** The primers used in RT-qPCR analysis.

| **Gene ID** | **Symbol** | **Primer** | **Sequences (5'-3')** | **Target products (bp)** |
| --- | --- | --- | --- | --- |
| RA291_13075 | *frdB* | RA291_13075-F | CAAGTGCATCGAGTGCTTC | 173 |
|  |  | RA291_13075-R | CCTGTGCGGTTCGTTTCC | 173 |
| RA291_06250 | *meaA* | RA291_06250-F | ACCGACATCCCCGTCATC | 157 |
|  |  | RA291_06250-R | CGAGGTGGTTCGCTTTCC | 157 |
| RA291_03975 | *monE* | RA291_03975-F | GCACTACGGCTACTGGTTCG | 79 |
|  |  | RA291_03975-R | CATCTCGTCGGTCATCTGG | 79 |
| RA291_06245 | *ccrA2* | RA291_06245-F | CAGACCCAGGACCCCAAGGA | 235 |
|  |  | RA291_06245-R | AGTGCGAGCCGATGATGC | 235 |
| RA291_30975 | *fadB* | RA291_30975-F | CCGTGGTGGAGAACGAGCAG | 99 |
|  |  | RA291_30975-R | TGGAGGAGGTGTTGGAGGC | 99 |
| RA291_03895 | *monD* | RA291_03895-F | CATCCCGAGCAGAAGCAG | 143 |
|  |  | RA291_03895-R | AACCTGTGGCCGTTCATG | 143 |
| RA291_26140 | *fabH* | RA291_26140-F | CCGAGTCCTGGGAGGTGTA | 178 |
|  |  | RA291_26140-R | GCCTGGTGCGGAATGAAG | 178 |
| 16S rRNA | *16S rRNA* | 16S rRNA-F | CAAGGCTAAAACTCAAAGGAA | 191 |
|  |  | 16S rRNA-R | AACCCAACATCTCACGACAC | 191 |
